# Most commonly used vaping brands by 18–25 year old young adults in Texas—Spring 2023

**DOI:** 10.1371/journal.pone.0300454

**Published:** 2024-05-31

**Authors:** Caroline North, Alexandra Loukas, Melissa B. Harrell, Keryn E. Pasch, C. Nathan Marti, Anna V. Wilkinson

**Affiliations:** 1 Department of Kinesiology and Health Education, The University of Texas at Austin, Austin, TX, United States of America; 2 Department of Epidemiology, UTHealth Houston School of Public Health, Austin, TX, United States of America; NYU Grossman School of Medicine: New York University School of Medicine, UNITED STATES

## Abstract

**Purpose:**

We aimed to determine (1) the most commonly used brands of electronic vaping products (EVPs) by young adults in Texas during Spring 2023, and (2) if brand preferences differ by sociodemographic characteristics, current cigarette smoking, and current cannabis vaping.

**Method:**

Participants were 2,491 18-25-year-olds (Mean age = 20.6; 62.9% female; 29.7% sexual gender minority; 35.9% non-Hispanic White, 45.0% Hispanic/Latino, 3.5% non-Hispanic Black, 11.6% non-Hispanic Asian, and 4.0% two or more races or another race/ethnicity) enrolled in 21 Texas colleges during February–March 2023 who used EVPs in the past 30-days.

**Results:**

Esco Bar was the most popular EVP brand (32.5%), followed by Elf Bar (19%), Vuse (10.1%), and all other brands were used by < 10% of participants. Nearly 20% of participants reported not having a usual brand. Participants who used Esco Bar, Elf Bar, and Puff Bar were younger (i.e., 18–20 years old), female, and Hispanic/Latino. Vuse, JUUL, and Smok were used by participants who were older (i.e., 21–25 years old), male, non-Hispanic white, used EVPs daily, and currently smoked cigarettes.

**Conclusion:**

The present study extends prior research by providing contemporary data on young adult EVP brand preferences in Texas during Spring 2023. Many of the brands commonly used by young adults (e.g., Esco Bar, Elf Bar) are not currently authorized for marketing or sale by the Food and Drug Administration. Findings underscore a need for additional enforcement efforts that prohibit the distribution and sale of these products to, in turn, prevent EVP use among young adults.

## Introduction

In recent years there has been a proliferation of electronic vaping products (EVPs) on the retail market in the United States (U.S.) [1]. Grand View Research reported that the U.S. EVP market size is expected to grow from $6.09 billion in 2020 to $40.25 billion by 2028 [2]. The increasing popularity of EVPs, in addition to the significant increases in nicotine strength across all EVPs in the U.S. [3], is particularly concerning given that many EVPs do not have authorization from the Food and Drug Administration (FDA) to be marketed or sold [4, 5]. As of 2020, young adults (18–25 years old) had the highest prevalence of EVP use among all age groups [6] and compared to older adults, young adults had the greatest increase in EVP use between 2019–2021 [7]. However, the most recent studies characterizing EVP brands were conducted between 2017 and 2020, and brands have evolved considerably since then, making it challenging to know what brands of EVPs young adults currently use [8]. Understanding the most common brands of EVPs used by young adults can provide greater insight into young adult EVP preferences and inform strategies to prevent access to, and use of, unregulated EVP brands.

Across multiple, nationally-representative, cross-sectional studies conducted between 2017–2020, JUUL remained the most popular brand of EVP used by adolescents and young adults [9–13]. During the same period, SMOK, Bo, Phix, Suorin, and Vuse also were identified as commonly used EVP brands among adolescents and young adults nationwide [11, 12]. Another nationally-representative study conducted in 2020 found that, among adolescents who ever used EVPs, the most popular brands were Puff Bar, VGOD, MyBlu, Phix, Stiizy, Mojo, Posh, Fogg, Halo, and Unicorn [11]. Although the last published study of young adults was conducted in 2020, recent data on adolescents from the 2022 National Youth Tobacco Survey showed that middle/high school students reported current use (i.e., used in the past 30 days) of Puff Bar, Vuse, Hyde, and SMOK [14] suggesting continued changes in brand preferences across time. Further, a national study of EVP unit sales across the U.S. (though not stratified by age) indicated that the top-selling EVP brands as of December 2022 were Breeze, Smok, Elf Bar, JUUL, NJOY, and Vuse [8]. As these studies show, brand preferences differ across time and age groups. However, it remains unclear what brands of EVPs young adults have been using since 2020. Understanding the brands young adults are using can inform FDA regulatory and enforcement efforts. For example, although FDA has begun to issue warning letters [15] and Marketing Denial Orders (MDOs) [16] to some EVP manufacturers to remove unauthorized products from the marketplace, their brands (e.g., Esco Bar, Elf Bar) continue to be available for sale. If young adults report using these brands, then FDA has critical, empirical evidence it needs to support and pursue additional enforcement efforts.

The present study aimed to fill the gap in the literature by examining brand preferences among a large sample of college students in Texas in Spring 2023. Although prior research has primarily assessed EVP brand use nationally, there is a need for regional surveillance, especially given that there are likely differences across states in how these products are marketed [17]. Moreover, there is a need to examine brand preferences among college students as they comprised 38% of the young adult population in 2021 [18] and Texas has the second-largest population of college students in the U.S. [19], making this population an important target for research and intervention. Using data from college students reporting past 30-day EVP use, we answered the following questions: (1) what are the most commonly used brands of EVPs by young adults in Texas during Spring 2023, and (2) do brand preferences differ by participant age, sex, sexual and gender minority status, race/ethnicity, daily EVP use, current cigarette smoking, and current cannabis vaping? Findings from this study will provide contemporary data on EVP brand use by young adults in Texas and inform public health efforts aiming to prevent access to, and ultimately use of, unauthorized/unregulated EVPs.

## Method

### Participants

Participants were a convenience sample of 2,491 18-25-year-old (Mean age = 20.6 [*SD* = 1.8]) students enrolled in 21 Texas colleges during February–March 2023 who used EVPs in the past 30 days. The students were 62.9% female; 29.7% sexual and gender minority (SGM; identifies as non-heterosexual and/or non-cis gendered); 35.9% non-Hispanic White, 45.0% Hispanic/Latino, 3.5% non-Hispanic Black, 11.6% non-Hispanic Asian, and 4.0% two or more races or another race/ethnicity.

### Procedures

The current sample was drawn from a larger cross-sectional sample of students enrolled in one of 21 colleges (6 two-year and 15 four-year; 3 private and 18 public) in Texas. A total of 371,826 prospective participants were recruited via email from February 28, 2023—March 27, 2023, to participate in an anonymous online survey regarding tobacco use, and 17,555 (4.7%) completed the survey after giving written informed consent. Participants who completed the survey were entered into a drawing for one of 50 $20 gift cards. Of the 17,555 students, 2,540 were 18–25 years old and used EVPs in the past 30 days. Of these, 49 participants were excluded due to incomplete data on focal variables (e.g., what brand they currently used), resulting in a sample size of 2,491. All study procedures were approved by the university Institutional Review Board (#2015080046).

### Measures

#### Sociodemographics

Age, sex, sexual and gender minority (SGM) status, and race/ethnic identity (non-Hispanic white, Hispanic/Latino, non-Hispanic Black, non-Hispanic Asian, another), were assessed. The SGM status variable was derived by combining two items, one that assessed sexual identity (i.e., identifying as heterosexual, gay, lesbian, bi, other) and the other assessing gender identity. Those who endorsed a non-heterosexual identity and/or identified as a gender identity other than cis-gendered (i.e., transgender, non-binary, genderqueer) were coded as being SGM. The race/ethnic identity variable was computed by combining two items, one that assessed race and the other that assessed Hispanic/Latino ethnicity. The ‘another’ category was computed by combining those who identified as American Indian or Alaskan Native, Native Hawaiian or other Pacific Islander, an unlisted race/ethnicity, and those who endorsed two or more race/ethnicity.

#### Current tobacco and cannabis use

Participants were asked about their past 30-day EVP use, cigarette use, and cannabis vaping. Current (i.e., past 30-day) EVP use was assessed with the following: “*During the past 30 days*, *how many days did you use ENDS (e*.*g*., *e-cigarettes*, *vape pens*, *e-hookahs*, *vape pods)*?” Current EVP use was recoded into a binary variable (0 days = 0 and 1+ days = 1) and was also recoded to indicate daily EVP use (1-29/30 days = 0 and 30/30 days = 1). Current cigarette smoking was assessed with: “*During the past 30 days*, *how many days did you use conventional/ combustible cigarettes*?” and current cannabis vaping was assessed with: “*During the past 30 days*, *how many days did you use cannabis*, *marijuana*, *or THC products*, *in the following products*? *Vaped*, *using marijuana concentrates*, *marijuana waxes*, *THC*, *or hash oils*?” Current cigarette smoking and current cannabis vaping were also dichotomized (0 days = 0 and 1+ days = 1).

#### EVP brand use

Participants indicating they used EVPs in the past 30 days were asked “*Which specific brand of e-cigarette/vaping device do you currently use most often*?” Response options included: Apollo, Aspire, BIDI Stick, Blu, Breeze (Breeze Pro of Breeze Plus), Elf Bar, Esco Bar, Geek Bar, Geek Vape, GreenSmoke, Hyde, JUUL, KangerTech, KangVape (including ONEE), Logic, Mr. Fog, NJOY, Posh, Puff Bar, Smok (including Nord, Novo), Suorin (including Air, Drop), VaporFi, and Vuse. Participants also were given the option to indicate “*I don’t have a usual brand*” or “*Other brand (please specify on the next page)*.” From the qualitative fill-in responses, an additional 74 unique brands were identified, providing a total of 99 unique brand categories. These 99 brands were then condensed into eight distinct categories based on the brands that were most highly endorsed by participants (i.e., each chosen brand was endorsed by > 1% of participants). Brands endorsed by less than 1% of participants were collapsed into a single category indicating “Other Brand.” Thus, eight brand categories were extracted; six that characterized unique EVP brands (Esco Bar, Elf Bar, Vuse, JUUL, Puff Bar, Smok), one “Other brand” category, and one for “No Usual Brand.”

### Data analysis

**Fig 1:**
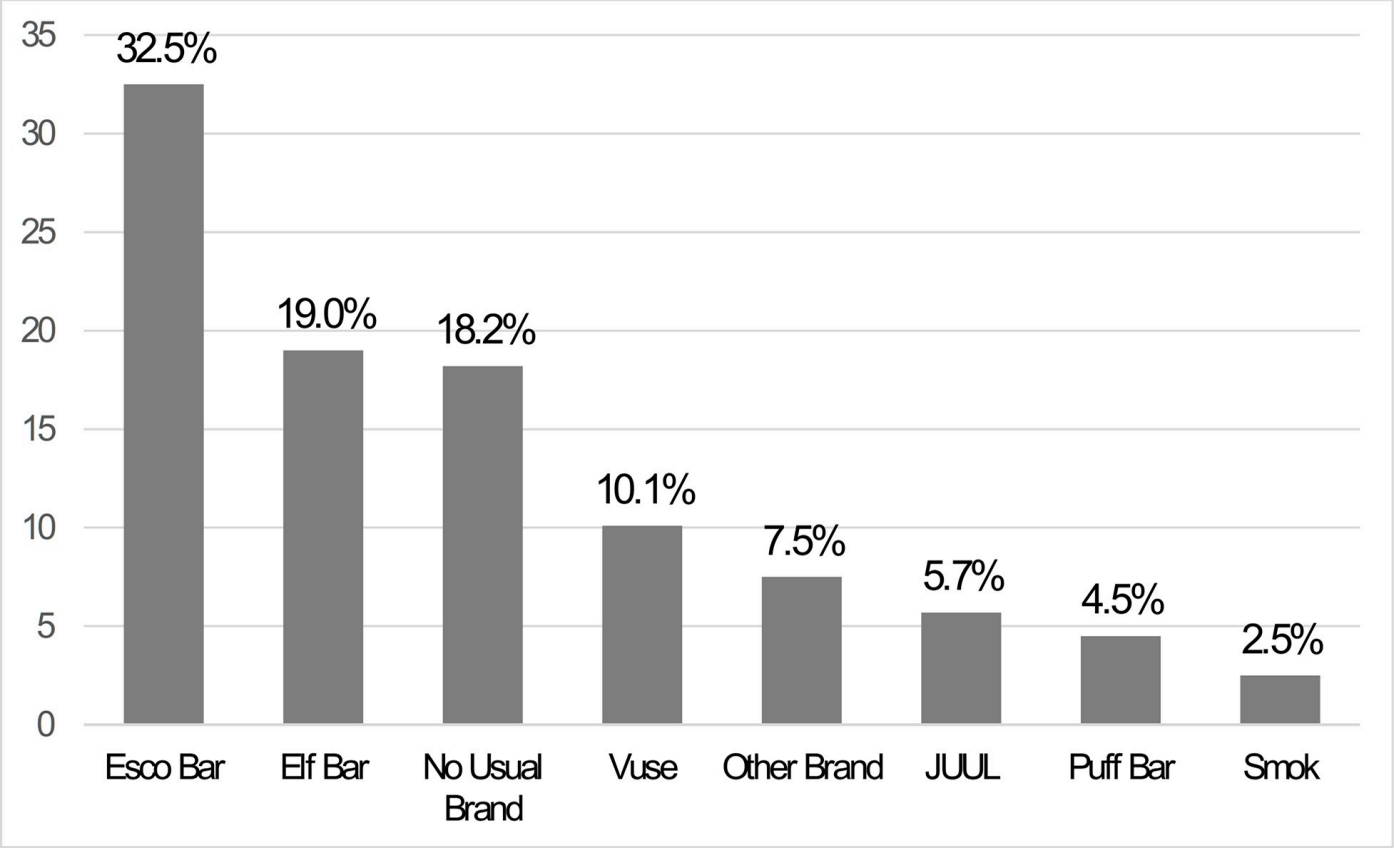
Prevalence of use for each of the most commonly used brands of EVPs among young adults in Texas who used EVPs in the past 30 days (n = 2,491) during Spring 2023.

EVP brand frequencies were used to assess which brands were used most often in the past 30 days, by brand category (Fig 1). To determine if brand preferences differed by sociodemographic characteristics (i.e., age, sex, SGM identity, race/ethnic identity), daily EVP use, current cigarette smoking, and current cannabis vaping (Table 1), proportions, and 95% confidence intervals associated with each proportion were calculated. If the confidence intervals did not overlap, the estimates were considered significantly different from each other at *p* < .006 [20].

**Table 1:** Differences in EVP brand use by sociodemographic factors, current tobacco and cannabis vaping, among young adults in Texas who used EVPs in the past 30 days (n = 2,491) during Spring 2023.

	Esco Bar (n = 810)	Elf Bar (n = 473)	Vuse (n = 252)	JUUL (n = 141)	Puff Bar (n = 111)	Smok (n = 63)
	% (95%CI)	% (95%CI)	% (95%CI)	% (95%CI)	% (95%CI)	% (95%CI)
**Age**						
18–20	54.1(50.6, 57.5) ^a^	50.5(45.9, 55.1) ^a^	50.0(43.7, 56.3) ^a^	33.3(25.6, 41.8) ^b^	50.5(40.8, 60.1) ^ab^	42.9(30.5, 56.0) ^ab^
**Sex**						
Male	30.6(27.5, 33.9) ^c^	41.2(36.8, 45.8) ^ab^	37.3(31.3, 43.6) ^abc^	44.0(35.6, 52.6) ^ab^	27.9(19.8, 37.2) ^ac^	52.4(39.4, 65.1) ^b^
**SGM Status**						
SGM	30.4(27.3, 33.7)	32.1(27.8, 36.5)	22.7(17.7, 28.4)	20.7(14.3, 28.4)	30.6(22.1, 40.2)	31.7(20.3, 45.0)
**Race/Ethnicity**						
White	33.3(30.1, 36.7) ^a^	37.8(33.5, 42.4) ^a^	57.9(51.6, 64.1) ^b^	36.9(28.9, 45.4) ^a^	13.5(7.8, 21.3) ^c^	58.7(45.6, 71.0) ^b^
Hispanic/Latino	47.2(43.7, 50.7) ^c^	37.5(33.0, 42.0) ^ab^	31.3(25.7, 37.5) ^a^	47.5(39.1, 56.1) ^bc^	73.0(63.7, 81.0) ^d^	28.6(17.9, 41.4) ^ab^
Black	3.7(2.5, 5.2)	2.5(1.3, 4.4)	2.8(1.1, 5.6)	2.9(0.8, 7.1)	3.6(1.0, 9.0)	1.6(0.04, 8.5)
Asian	11.2(9.1, 13.6)	17.8(14.4, 21.5)	4.8(2.5, 8.2)	9.(5.0, 15.3)	6.3(2.6, 12.6)	7.9(2.6, 17.6)
Another Race/Ethnicity	4.6(3.2, 6.2)	4.4(2.8, 6.7)	3.2(1.4, 6.2)	3.5(1.2, 8.1)	3.6(1.0, 9.0)	3.2(0.4, 11.0)
**Daily EVP Use**						
Daily Use (30/30 days)	29.0(25.9, 32.3) ^a^	35.1(30.8, 39.6) ^a^	49.2(42.9, 55.6) ^b^	29.1(21.7, 37.3) ^a^	7.2(3.2, 13.7) ^c^	66.7(53.7, 78.0) ^b^
**Current Smoking**						
Past 30 Day Use	26.9(23.8, 30.0) ^a^	27.7(23.7, 32.0) ^a^	29.0(23.4, 35.0) ^ab^	34.3(26.5, 42.8) ^a^	15.3(9.2, 23.4) ^b^	25.4(15.3, 37.9) ^ab^
**Current Cannabis Vaping**						
Past 30 Day Use	41.9(38.4, 45.3)	35.3(31.0, 39.8)	40.5(34.4, 46.8)	30.5(23.0, 38.8)	35.1(26.3, 44.8)	34.9(23.3, 48.0)

***Note*.** The categories “No Usual Brand” and “Other Brand” were not included because they did not signify a specific brand.

SGM = sexual gender minority (i.e., identifies as non-heterosexual and/or transgender).

The same superscript denotes no significant difference while different superscripts within a row denote a significant difference.

## Results

**Fig 2:**
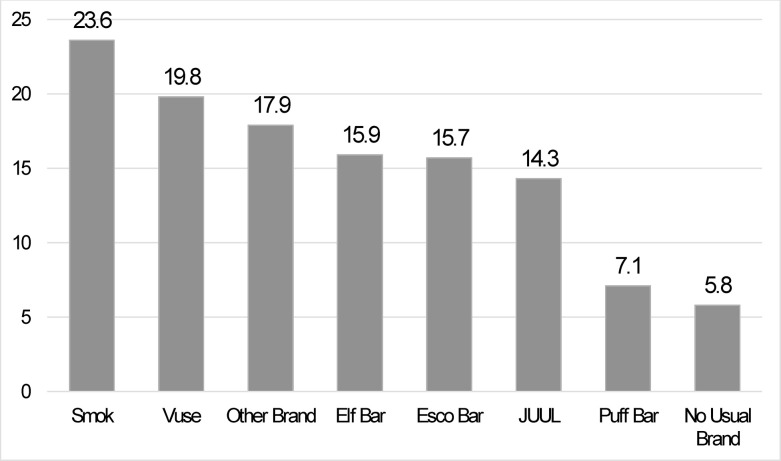
Mean number of days used in the past 30 for each of the most commonly used brands of EVPs among young adults in Texas who used EVPs in the past 30 days (n = 2,491) during Spring 2023.

As shown in Fig 1, nearly a third of the sample endorsed Esco Bar as the EVP they used the most in the past 30 days, followed by Elf Bar. The third most commonly used EVP brand was Vuse, followed by ‘other’ brand, JUUL, Puff Bar, and then Smok. Of note, over 18% of young adults who used EVPs in the past 30 days indicated they did not have a usual brand. As shown in Fig 2, participants with no usual EVP brand used an EVP on fewer days out of the past 30 compared to those with an identified EVP brand. Although the use of Smok was endorsed by the least number of participants, those who used Smok used on the greatest number of days in the past 30 (*M* = 23.6).

Table 1 displays sociodemographic differences across each EVP brand and differences in product use. Older young adults (aged 21–25) were more likely to endorse using JUUL and Smok compared to younger young adults (aged 18–20), and a larger proportion of younger young adults (aged 18–20) used Esco Bar and Elf Bar. Female participants were more likely to use Esco Bar and Puff Bar, while males were more likely to use Smok. A larger proportion of White participants used Vuse and Smok whereas a larger proportion of Hispanic/Latino participants endorsed using Puff Bar. No other significant differences were present across the other race/ethnicity groups or by SGM status.

Regarding differences by daily EVP use status, participants who used Vuse and Smok were more likely to endorse daily EVP use than their counterparts and those who used Puff Bar were least likely to use EVPs daily. Finally, a larger proportion of those who used JUUL endorsed current cigarette smoking compared to their counterparts, while the smallest proportion of current cigarette smoking was seen among those who used Puff Bar. No differences across EVP brands were observed for current cannabis vaping status. However, it is important to note that the prevalence of current cannabis vaping across each EVP brand ranged from 30.5% to 41.9%, compared to 15.3% to 34.3% for cigarette smoking across each EVP brand.

## Discussion

The present study extends our limited understanding of EVP brand preferences by identifying the most commonly used brands of EVPs by 18-25-year-old young adults in Texas in Spring 2023. Consistent with prior surveillance research conducted before or in 2020, brands including Puff Bar, Vuse, JUUL, and SMOK were among the most commonly used EVP brands by young adults in this sample [9–14]. However, these brands were not the most popular- Esco Bar and Elf Bar were the two most commonly used EVP brands by young adults in Texas in Spring 2023, indicating that population-based brand preferences can and do change over a relatively short period of time. Over half (51.1%) of the participants reported currently using Esco Bar or Elf Bar, which is troubling given that neither brand is authorized for marketing and/or sale by the FDA [21]. In fact, all of the identified brands in this study, with the exception of JUUL, have received FDA MDOs and/or FDA warning letters for the unauthorized selling of these products [21], underscoring the need for enhanced and stronger enforcement of FDA regulatory authority.

The present study also extends prior research by identifying differences in sociodemographic characteristics, current cigarette smoking, and current cannabis vaping by EVP brand used. A larger proportion of participants using Esco Bar, Elf Bar, and Puff Bar were younger (i.e., 18–20 years old), female, and identified as Hispanic/Latino compared to young adults who used Vuse, JUUL, and Smok. Compared to the other identified brands, young adults who used Esco Bar, Elf Bar, and Puff Bar used their EVP a fewer number of days in the past month, on average, and were less likely to use EVPs daily. Thus the findings from the present study suggest that participants who used Esco Bar, Elf Bar, and Puff Bar may not have been established in their EVP use because they were less likely to use EVPs daily, an indicator of established use [22]. Experimental and non-established use of EVPs is typical for young adult college students [23]. Although those who used Esco Bar and Elf Bar were less likely to use EVPs as frequently as those who use other brands (i.e., JUUL, Vuse, Smok), it is important to note that participants using Esco Bar and Elf Bar indicated that they used their EVP on at least half of the past 30 days on average, which is considered moderate use [24]. This is troubling given that Esco Bar and Elf Bar have high concentrations of nicotine, up to 50 mg/mL (i.e., 5%), and recent research indicates that young adults may use EVPs in a habitual (i.e., automatic and unconscious) manner, which is likely due to the ease and concealability of EVPs [25]. Habitual use of high-concentration EVPs may increase the risk for the development of nicotine dependence and potentially lifelong nicotine use [25]. Thus, future research should track young adults who use brands like Esco Bar and Elf Bar into adulthood to determine if the use of these EVP brands leads to nicotine dependence.

In contrast to participants who used Esco Bar, Elf Bar, and Puff Bar, those who used Vuse, JUUL, and Smok were more likely to be older (i.e., 21–25 years old), male, non-Hispanic white, use EVPs daily, and currently smoke cigarettes. Those who use Vuse, JUUL, and Smok are likely not “light”/experimental EVP users but rather appear to be more frequent/regular EVP users because they endorsed using EVPs on a greater number of days in the past 30 on average (*M* = 19.8, *M* = 14.3, *M* = 23.6, respectively; see Fig 2) and a large proportion reported using EVPs daily (49.2%, 29.1%, 66.7%, respectively; see Table 1) [24]. Moreover, those who use Vuse, JUUL, and Smok may be using their EVP brand in place of, or to supplement, cigarettes where smoking is not allowed [26], such as on college campuses [27], or to cut down or quit cigarettes. Longitudinal evidence indicates that adults who are dual users of cigarettes and EVPs are less likely than those who use one product only to discontinue the use of either or both products in the future [28, 29], increasing the risk for higher nicotine exposure, negative health effects, and decreased likelihood of cessation [30, 31]. Further, the nicotine concentrations of these products are especially high, increasing the potential for nicotine dependence [32–34]. Taken together, these findings may suggest that young adults who use Vuse, JUUL, and Smok may have already developed symptoms of nicotine dependence [35], however, additional research is needed to determine if EVP brand preferences are associated with levels of nicotine dependence.

It should be noted that only 4.5% of young adults reported using Puff Bar most frequently in the past 30 days, and these participants were the least likely to report daily EVP use or current cigarette smoking. The low prevalence of Puff Bar use was surprising given prior research showing that it was one of the most popular EVP brands among adolescents as recently as 2022 [11, 14]. However, Puff Bar recently stopped selling nicotine-based EVPs and now sells what they are calling “Zero Nicotine” and Delta-8 THC products [36]. It is therefore uncertain whether young adults who used Puff Bar were vaping nicotine or THC in their EVP during the time of data collection. Puff Bar exemplifies the rapidly changing EVP landscape and highlights the need for future research that captures the substances that young adults are using in their devices. In the present study, 30–42% of participants also currently vaped cannabis and there were no differences in the prevalence of cannabis vaping across EVP brands. The high prevalence of cannabis vaping among this young adult population also warrants additional research and raises questions about the nature of dual nicotine and cannabis vaping, and the long-term consequences [37].

The strengths of the present study include a large ethnically diverse sample of young adults recruited from 21 colleges in Texas and the open-ended response measurement of current EVP brands used. The present study also has limitations including the lack of generalizability to national samples of young adults. However, these data provide a regional comparison with other nationally-representative studies, and Texas has the second largest population of college students in the U.S. making this a valuable sample for understanding young adult college students’ EVP brand preferences. Additional surveillance research should be conducted to determine if EVP brand preferences differ in other states and/or regions of the U.S.

In sum, findings provide contemporary data regarding current EVP brand preferences held by young adults in Texas during Spring 2023. Alarmingly, young adults use many EVPs that are not authorized for marketing or sale by the FDA indicating a need for additional comprehensive policies and enforcement efforts. Informing young adults that many of the EVP brands they use are not currently regulated or authorized by the FDA may prove useful in deterring use. Future research should continue to surveil EVP brand preferences among young adults, the population that has the highest prevalence of EVP use [6], as the EVP landscape continues to change. Precise information about the EVP brands young adults are using, as well as the contents of their devices (e.g., the concentration of nicotine, cannabis, etc.), is needed to better inform FDA regulatory and enforcement efforts aiming to decrease the use of unauthorized and unregulated EVPs.
